# Universal digital high-resolution melt: a novel approach to broad-based profiling of heterogeneous biological samples

**DOI:** 10.1093/nar/gkv1083

**Published:** 2015-10-17

**Authors:** Stephanie I. Fraley, Justin Hardick, Billie J. Masek, Pornpat Athamanolap, Richard E. Rothman, Charlotte A. Gaydos, Karen C. Carroll, Teresa Wakefield, Tza-Huei Wang, Samuel Yang

*Nucl. Acids Res*. (2013) 41 (18): e175. doi: 10.1093/nar/gkt684

The authors wish to correct the full name of Billie Jo Masek to Billie J. Masek.

